# Antifungal Activity of Polymethoxylated Flavonoids (PMFs)-Loaded Citral Nanoemulsion against *Penicillium italicum* by Causing Cell Membrane Damage

**DOI:** 10.3390/jof8040388

**Published:** 2022-04-11

**Authors:** Long Guo, Yi Li, Xiaoxue Mao, Rui Tao, Boyun Tao, Zhiqin Zhou

**Affiliations:** 1 College of Horticulture and Landscape Architecture, Southwest University, Chongqing 400716, China; gl1466878376@email.swu.edu.cn (L.G.); m_liyi@163.com (Y.L.); mofei770501@163.com (X.M.); tr858618941@163.com (R.T.); yun7412369@email.swu.edu.cn (B.T.); 2 Key Laboratory of Horticulture Science for Southern Mountainous Regions, Ministry of Education, Chongqing 400715, China; 3 The Southwest Institute of Fruits Nutrition, Banan District, Chongqing 400054, China

**Keywords:** antifungal mechanism, citrus blue mold, membrane integrity, membrane permeability, *P. italicum*

## Abstract

A major citrus postharvest pathogen, *Penicillium* *italicum* (*P. italicum*), causes substantial economic losses in citrus. In this study, a citral nanoemulsion containing polymethoxylated flavonoids (PMFs), the antimicrobial compounds from citrus, was prepared. The antifungal activity and potential antifungal mechanisms of the nanoemulsion against *P. italicum* were evaluated. The results showed that the growth of *P. italicum* was effectively inhibited by the nanoemulsion, with a minimum inhibitory concentration (MIC) and minimum fungicidal concentration (MFC) of 62.5 and 250 mg L^−1^, respectively. The nanoemulsion significantly inhibited spore germination and mycelial growth, and it altered the morphology of *P. italicum*. In addition, the permeability of the cell membrane increased with increasing nanoemulsion concentrations, as evidenced by a rapid rise in extracellular electric conductivity and stronger red fluorescence from mycelia (propidium iodide staining). Compared with the control, the nanoemulsion treatment induced a decrease in total lipid and ergosterol contents in *P. italicum* cells by 64.61% and 60.58%, respectively, demonstrating that membrane integrity had been disrupted. The results indicated that the PMFs-loaded nanoemulsion exerted antifungal activity against *P. italicum* by disrupting cell membrane integrity and permeability; such a nanoemulsion may be used as a potential fungicide substitute for preservation in citrus fruits.

## 1. Introduction

Citrus is among the most important fruit crops, whose producing area in the world extends approximately 40° north in latitude to 40° south in latitude from the equator [1,2]. According to the Food and Agriculture Organization (FAO), more than 140 million tons of citrus was harvested in 2019 [3]. However, pathogens such as *Penicillium*, *Geotrichum citriaurantii*, and *Alternaria alternata* damage citrus crops severely, causing postharvest losses of massive proportions [4,5]. In China, *Penicillium italicum* one of the most prominent causes of blue mold disease on citrus fruit, causing a postharvest loss of 20%–50% [6]. As a result, synthetic fungicides such as imazalil, prochloraz, or thiabendazole have been widely used on citrus fruits to prevent blue mold disease [7]. However, there are concerns with the use of synthetic fungicides because they may cause public health issues by leaving toxic residues in fruits and the environment [8,9]. On the basis of the above issues, numerous studies have explored natural phytochemicals that have antifungal properties and no negative effects.

Polymethoxylated flavones (PMFs) are a group of flavonoids mainly derived from citrus peel and have numerous strong biological activities, including anti-inflammatory [10], antioxidant [11], antimicrobial [12], and anticancer [13]. Citral, an aldehyde mixture of geranial and neral, is widely found in many citrus essential oils and has been shown to have strong antifungal activity against the pathogens *Fusarium*, *Botrytis*, *Penicillium*, and *Aspergillus* [14,15,16], which are generally recognized as safe (GRAS) for human health and the environment [17]. Researchers have documented that PMFs and citral have the ability to inhibit fungal growth and could be used as antifungal agents in the food industry [18,19]. In addition, the potential antifungal mechanisms of PMFs and citral both exhibit the direct inhibition of pathogens such as through inhibiting mycelial growth, destroying the integrity and permeability of cell membranes, and releasing cellular components [18,20]. However, the high volatility of citral and the poor water solubility of PMFs are the main limiting factors for maximizing their antifungal effects in practice. Herein, we focused on using nanotechnology to enhance their characteristics and further broaden their applications. In this study, we selected an oil-in-water nanoemulsion system for the encapsulation of PMFs and citral to solve the aforementioned problems.

Combining antimicrobials enhances antimicrobial activity and reduces the emergence of resistant strains and the amount of fungicide required [21,22]. Research has shown that the combined effects of certain EOs can increase fungistatic activity, reducing the ability of pathogens to develop resistance to multiple EOs [23,24]. In addition, combinations of volatiles and nonvolatile antimicrobial components enhanced the antimicrobial efficacy and maintained the long-term potency of antimicrobial agents of most pathogens [25,26,27]. Considering that the volatility of citral remains a limitation to the antifungal activity of a citral nanoemulsion when used in citrus fruit storage under standard conditions, we attempted to create novel antifungal agents against *P. italicum* by adding nonvolatile constituents, PMFs, to a citral nanoemulsion.

Although the antifungal activities of citral and PMFs have been widely studied, the combination of PMFs with citral and their combined effect on *P. italicum* has not been investigated. Thus, this study aimed to develop a novel nanoemulsion containing PMFs and citral by combining the positive effects of these two compounds and to evaluate the antifungal activities and potential antifungal mechanisms of PMFs-loaded citral nanoemulsions against *P. italicum*.

## 2. Materials and Methods

### 2.1. Fungi and Chemicals

Isolation and purification of *P. italicum* were performed at the Key Laboratory College of Horticulture and Landscape Architecture, Southwest University (Chongqing, China). The fungi were inoculated on PDA and incubated at 28 °C for 3 days before use.

PMFs were purchased from Shaanxi Huike Plant Development Co., Ltd (Shaanxi, China). Citral was purchased from Aladdin Reagent Database Co. (Shanghai, China). Caprylic/capric triglyceride (GTCC) was supplied by Shanghai Yuanye Biotechnology Co., Ltd (Shanghai, China). Tween 80 (TW80) was obtained from Kelon Chemical Reagent Factory (Chengdu, China). The fluorescent probe propidium iodide (PI) was obtained from Sangon Biotech Co., Ltd. (Shanghai, China).

### 2.2. Analysis of the Components of PMFs Dissolved in Citral by UPLC–PDA

The chemical profile of the PMFs dissolved in citral was analyzed by UPLC–PDA (Waters, Milford, MA, USA) [28]. Firstly, 30 mg of PMFs was weighed and dissolved in 1 g of citral, filtered with 0.22 μm organic membrane, and diluted with methanol 30 times before UPLC–PDA analysis (Waters Corporation, MA, USA). The determination of PMFs dissolved in citral was based on a UPLC BEH C_18_ column (2.10 × 100 mm, 1.70 μm) at a column temperature of 40 °C and a flow rate of 0.4 mL/min. The mobile phase included solvent A (water containing 0.01% formic acid) and solvent B (methanol), and the absorption wavelength was set to 330 nm for PMFs.

### 2.3. Nanoemulsion Preparation

With some alterations to a previously reported method [29], nanoemulsions were prepared. Initially, 30 mg PMFs were dissolved in 1 g citral and then mixed with 0.5 g GTCC and 3 g TW 80 after filtration through a 0.22 μm organic membrane. Then, after the mixture was stirred in a magnetic stirrer (DF-II, Jintan Youlian Instrument Research Institute, China) at a speed of 2600 r/min for 30 min, distilled water was added dropwise with continuous stirring. After that, the obtained emulsions were ultrasonicated for 5 min at 60 amplitude with an ultrasonicator (KQ-500DE; Kunshan Ultrasound Instrument Co., Ltd., Shanghai, China) to reduce the droplet size. The same technique was used to prepare nanoemulsions without PMFs. All nanoemulsions were then stored at 4 °C for subsequent analysis. For convenience, the citral nanoemulsion and PMFs-loaded citral nanoemulsion were labelled as the CT and PCT nanoemulsions, respectively. According to the initially prepared PCT nanoemulsion calculations, the citral concentration in the nanoemulsion was 20 g L^−1^ and the PMF concentration was 0.38 g L^−1^.

### 2.4. Characterization of Nanoemulsions: Particle Size, Polydispersity Index (PDI), and Zeta Potential

The zeta potential and particle size of nanoemulsions were determined with a dynamic-light-scattering Zetasizer (Malvern Instruments, Worcestershire, UK). Additionally, we evaluated the polydispersity index (PDI), which represented the particle size distribution of the nanoemulsion. All measurements were performed at room temperature (25 °C). The averages were based on 3 measurements per sample and 100 runs per measurement [30].

### 2.5. Determination of Minimal Inhibitory Concentration (MIC) and Minimum Fungicidal Concentration (MFC)

The MIC and MFC of the nanoemulsions against *P. italicum* were evaluated by a modified dilution method on 96-well plates [31]. Briefly, 100 μL of the mixture containing CT or PCT nanoemulsion and PDB was prepared, making the final citral concentrations range from 2000 to 62.5 mg L^−1^. Next, to each well, we added 100 μL of spore suspension (10^5^ CFU·mL^−^^1^), and they thoroughly mixed to obtain a citral concentration ranging from 1000 to 31.25 mg L^−1^. Afterwards, the sealed and cultured 96-well plates were incubated for 72 h at 28 °C. The positive control consisted of PDB mixed with the conidial suspension, and the negative control included only PDB. After incubation for 72 h, the MIC was defined as the minimum concentration without growth of *P. italicum*. Then, 100 μL mixture was removed from the wells in which no fungus grew and transferred to PDA for another 72 h of incubation. The MFC was defined as the minimum concentration without growth of *P. italicum* on the PDA plate.

### 2.6. Effect of Nanoemulsion on Spore Germination

The effect on spore germination by nanoemulsions in *P. italicum* was examined by microscopy observation [32]. Briefly, 2 mL of the mixture containing freshly prepared conidial suspension (10^7^ CFU mL^−1^) and PCT nanoemulsion was prepared in a glass tube, making a final citral concentration of 0, 1/2 MIC, MIC, and 2 MIC. Then, the sealed mixture was cultured at 28 °C. After 5, 12, and 24 h, 100 μL of each mixture was taken and placed in glass depression slides to observe spore germination under a microscope (OLYMPUS TH4-200, Olympus Optical Co., Ltd., Tokyo, Japan). When the length of a germ tube was longer than its length, the conidium was considered to have germinated.

### 2.7. Effect of Nanoemulsion on Mycelial Growth

An Oxford cup method [33] with some alterations was used to determine the effect of nanoemulsions on the mycelia growth of *P. italicum*. Briefly, a certain volume of PCT nanoemulsion was added to the sterilized PDA medium to make the final citral concentrations of 0, 1/2 MIC, MIC, and 2 MIC. After medium solidification, sterilized Oxford cups (φ 6 mm) were placed in the middle of each Petri dish (50 mm in diameter) and filled with 100 μL conidial suspension. Then, all sealed plates were incubated at 28 °C for one week. After 3 days of incubation, we began to measure and record the colony diameters of each group. On the basis of the following equation, the inhibitory rate of mycelial growth was calculated:

$$\mathrm{Inhibition}\;\mathrm{rate}\;\mathrm{of}\;\mathrm{mycelial}\;\mathrm{growth}\;\left(\%\right)=\frac{Dc-Dt}{Dc-Di}\times 100$$

In the equation, *Dc* (mm) means colony diameter of control groups, *Dt* (mm) means colony diameter of treatment groups, and *Di* (mm) means the diameter of the Oxford Cup.

### 2.8. Determination of cell Membrane Integrity and Permeability

#### 2.8.1. Morphological Observation

Scanning electron microscopy (SEM) was used to observe the effect of nanoemulsion treatment on the micromorphology and ultrastructure of *P. italicum*. Briefly, fungal cultures grown on the PDA plates for 5 days after treatment with various PCT concentrations (0, 1/2 MIC, MIC, and 2 MIC) were cut into segments of ~2 mm^3^ and fixed with a glutaraldehyde solution of 2.5% overnight at 4 °C. Then, the segments were washed three times with 0.1 mol L^−1^ phosphate buffer (pH = 7.2) for 15 min each and dehydrated with graded ethanol series (30, 50, 75, 85, and 100%) for 15 min. After drying, the tissues were vacuum plated with gold (10 KV, 220 s) and observed with a scanning electron microscope (Hitachi SU8020, Tokyo, Japan) [34].

#### 2.8.2. Propidium iodide (PI) Staining

PI staining used in [35] with some alterations was used to assess the damage of PCT nanoemulsion treatment on the cell membrane of *P. italicum*. Firstly, a mixture containing a certain volume of PCT and sterilized PDA was prepared, making the final concentration of citral reach 0, 1/2 MIC, MIC, and 2 MIC. Then, the mixture was poured into the Petri dish (50 mm in diameter). After cooling, 20 μL of spore suspension (10^6^ CFU mL^−1^) was spread on the surface of the PDA medium, and cover glass was inserted in the middle of the PDA medium at an oblique angle. After incubating at 28 °C for 3 days, the cover glass was transferred to a glass slide and 10 μL PI reagent was added for staining. After 15 min of darkness, the excess dye on the sample was removed with sterile water. Then, the samples were observed under a microscope (OLYMPUS TH4-200, Olympus Optical Co, Ltd., Tokyo, Japan).

#### 2.8.3. Extracellular Electric Conductivity

The extracellular conductivities of *P. italicum* mycelia treated with different concentrations of PCT (0, 1/2 MIC, MIC, and 2 MIC) were measured by an electric conductivity meter (Century Ark Technology Co. Ltd., Chengdu, China), according to the method previously described by Li et al. [25] with minor alterations. The electric conductivity was determined after incubation for 4, 8, 12, 24, 36, and 48 h, and the extracellular conductivity was expressed as the relative electric conductivity (*REC*, %).

$$\mathrm{Relative}\;\mathrm{electric}\;\mathrm{conductivity}\;\left(REC,\%\right)=\frac{R_{t}-R_{0}}{R_{k}-R_{0}}\times 100$$

In the formula, *R*_0_ is the electric conductivity of pure water (25 °C), *R_t_* is the electric conductivity of different treatments at different incubation times, and *R_k_* is the electric conductivity of the mycelia after boiling.

#### 2.8.4. Malondialdehyde (MDA)

The measurement of MDA content was carried out using the thiobarbituric acid (TBA) method previously described by Li et al. [36] with some alterations. Firstly, a mixture containing PCT nanoemulsion and sterilized PDA medium was prepared, making the final concentration of citral reach 0, 1/2 MIC, MIC, and 2 MIC. Then, the medium was poured into a Petri dish (90 mm in diameter). After medium solidification, a layer of cellophane was placed on it. Then, 100 μL of freshly prepared spore suspension (10^6^ CFU mL^−1^) was spread evenly on the cellophane, and it was incubated at 28 °C for 5 days. Following that, 0.1 g of mycelium cultured on cellophane was weighed and ground with 4 mL of 5% trichloroacetic acid (TCA) solution; after being centrifuged at 10,000× *g* for 10 min, 1 mL of the supernatant was mixed with 1 mL of 0.67% thiobarbituric acid (TBA) and boiled at 100 °C for 30 min. After cooling, the sample was centrifuged at 10,000× *g* for 10 min. Next, the OD values of the supernatants were measured at 450, 532, and 600 nm. The *MDA content* was calculated using the following equation:

$$MDA\;content\;(\mathrm{nmol}/g=\left[6.45\times \left(OD_{532}-OD_{600}\right)-0.56\times OD_{450}\right]\times k\times \frac{V_{t}}{m}$$

In the equation, *Vt* (mL) is the volume of the total extract solution, *k* is the dilution factor, and *m* (g) is the mass of fresh mycelia.

#### 2.8.5. Total Lipids and Ergosterol

The phosphoric acid–vanillin method was used to determine the total lipid content of *P. italicum* treated with different concentrations of PCT with minor alterations [37]. Using triglyceride as a standard, the lipid content (g kg^−1^) of the mycelium was determined on the basis of the calibration curve. The total ergosterol content of *P. italicum* cell membrane treated with different concentrations of PCT was determined using the HPLC method optimized and reported by Li et al. [38]. The results were expressed as g kg^−1^ mycelia dry weight. Each treatment was repeated three times.

### 2.9. Statistical Analysis

All data were analyzed and processed using the SPSS 25.0 statistical software. Analysis of variance was performed to analyze the significance among the different groups using Duncan’s multiple range test (*p* < 0.05) following one-way ANOVA.

## 3. Results

### 3.1. Analysis of the Components of PMFs Dissolved in Citral by UPLC–PDA

The components of PMFs dissolved in citral were analyzed (Table 1) by UPLC–PDA. Five PMF monomers were detected, among which nobiletin and tangeretin were the most prominent, accounting for more than 95% of the total.

### 3.2. Characterization of the Nanoemulsions

As shown in Table 2, during the 20-day storage at room temperature, the PCT size increased significantly from 17.08 to 26.86 nm. The PDI value (0.215) of the PCT in this study represented a narrow size distribution, indicating a good distribution of the PCT. Zeta potential is an important parameter for analyzing the stability of encapsulation systems. A high zeta potential creates a repelling force between particles, which increases the stability of nanoemulsions. In this study, the zeta potential of PCT went from a highly negative value (22.20 mV) to a moderately negative value (11.05 mV). Therefore, the PCT was assumed to have relatively good stability.

### 3.3. MIC and MFC of the CT and PCT Nanoemulsions against P. italicum

The determination of the MIC and MFC of the nanoemulsions against *P. italicum* were based on observations of *P. italicum* growth on 96-well plates (Table 3). After 72 h of incubation, it was observed that, for mycelial growth, the maximum concentration of the CT group was 62.5 mg L^−1^ and that of the PCT group was 31.25 mg L^−1^. After transferring the mixture from the 96-well plates to the PDA and culturing for another 72 h, mycelial growth was inhibited by the CT and PCT nanoemulsions of 250 mg L^−1^. Based on the definitions, the MFCs of both the CT and PCT nanoemulsions were 250 mg L^−1^, while the MIC of the CT nanoemulsion was 125 mg L^−1^ and that of the PCT nanoemulsion was 62.5 mg L^−1^. The results indicated that the PCT nanoemulsion showed more effective antifungal activity than the CT nanoemulsion.

### 3.4. Effect on Spore Germination

The inhibition effect of PCT on the spore germination of *P. italicum* in PDB was investigated. At 5, 12, and 24 h, the spore germination rates of CK were 4.91%, 53.45%, and 91.69%, respectively (Table 4). However, the percentages of germinated spore in the 1/2 MIC and MIC groups were significantly reduced. Furthermore, there was almost no spore germination in the 2 MIC group. This suggested that PCT significantly inhibited spore germination in a dose-dependent manner.

### 3.5. Effect on Mycelial Growth

**Figure 1:**
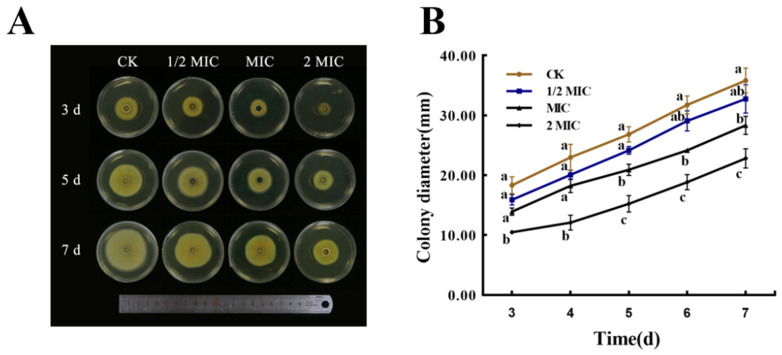
Effects of different concentrations of a PCT nanoemulsion on colony morphology and diameter of *P. italicum.* (**A**) Colony morphology at 3, 5, and 7 days. (**B**) Colony diameter from 3 to 7 days. Each value represents the mean ± SD. Means with different letters were significantly different at *p* < 0.05.

The mycelial growth of *P. italicum* was affected by the nanoemulsions, as shown in Figure 1. Compared with the control group, the treatment group had significant differences. On the same day, the diameter of the mycelial disk in the control group was larger than that of the treatment group, and the treatment group decreased with increased PCT concentration. Groups treated with low concentrations (MIC and 1/2 MIC) showed lower inhibition compared with those treated with high concentrations (2 MIC). The results indicated that the mycelial growth of *P. italicum* was effectively inhibited by the PCT nanoemulsions in a dose-dependent manner.

### 3.6. Micromorphological Analysis by SEM

**Figure 2:**
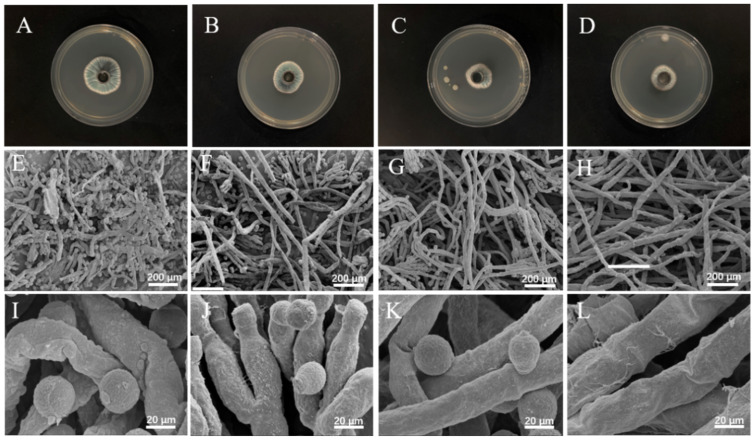
(**A**–**D**) Samples used for SEM observation. (**E**–**L**) Morphologies of the mycelia and conidia of *P. italicum* were observed by SEM after treatments with 0 (**E**,**I**), 1/2 MIC (**F**,**J**), MIC (**G**,**K**), and 2 MIC PCT nanoemulsions (**H**,**L**). Scale bar, 200 and 20 µm.

The morphological changes in spore germination in mycelia after being treated with PCT at different concentrations (0, 1/2 MIC, MIC, and 2 MIC) are shown in Figure 2. The mycelial disk of the control grown on PDA showed a blue color, with more mellow and healthy spores, as well as homogenous mycelia with normal morphology and more fluff (Figure 2A,E,I). By contrast, when exposed to PCT, the color of the mycelial disk, normal mycelial morphology, and spore germination were conspicuously changed. In those treated with PCT at 1/2 MIC and MIC, the color of the mycelial disk gradually changed from blue to white, the shape of the spores became abnormal and the number gradually decreased, and the mycelia gradually dried up (Figure 2B,C,F,G,J,K). When treated with PCT at 2 MIC, the color of the disk became completely white; the number of spores was extremely low; and the mycelia appeared shriveled, shrunken, and ruptured (Figure 2D,H,L). These results indicated that the PCT nanoemulsion inhibited *P. italicum* by affecting spore germination and destroying the structures of spores and mycelia.

### 3.7. Propidium iodide (PI) Staining

**Figure 3:**
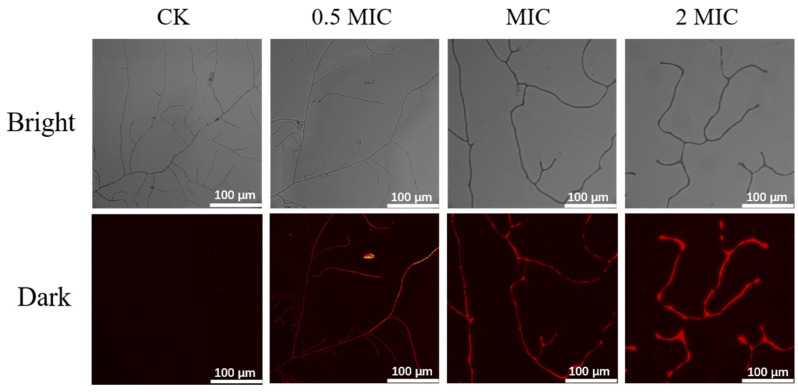
Mycelia morphology (in the bright field) and the corresponding PI staining images (in the dark field) of *P. italicum* treated with different concentrations of a PCT nanoemulsion. Scale bar, 100 µm.

PI is expelled from living cells actively, and its uptake reflects the integrity and permeability of the cell membrane during the late stages of apoptosis [35]. As shown in Figure 3, *P. italicum* mycelia displayed different states in the four groups after 2 days of incubation. Microscopic observations revealed that untreated mycelia had a visible linear shape and septa. The treatment groups appeared to shrivel and invaginate. In addition, mycelia exposed to nanoemulsions showed more red fluorescence under fluorescence irradiation, and the intensity of that fluorescence increased with the concentration of the PCT nanoemulsion, suggesting that the nanoemulsion profoundly damaged cell membranes.

### 3.8. Extracellular Conductivity

**Figure 4:**
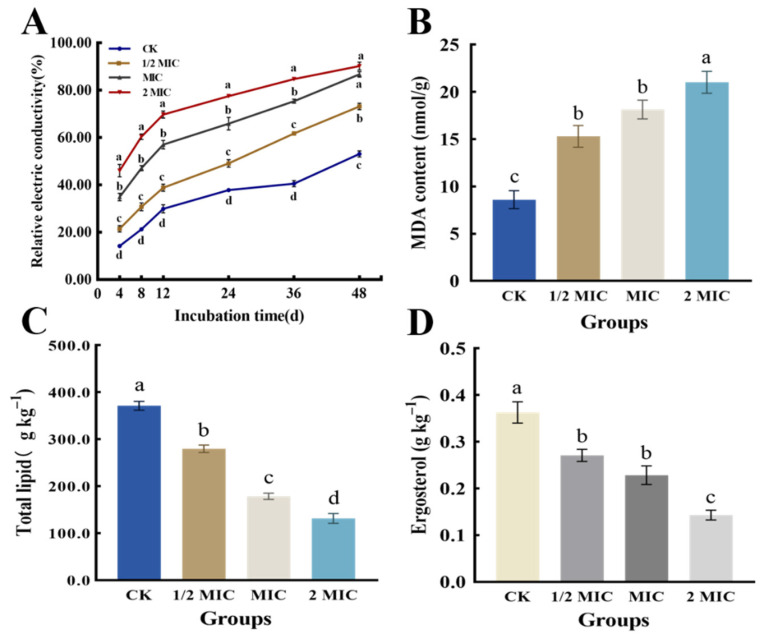
Effects of PCT nanoemulsions on the (**A**) relative electric conductivity and (**B**) MDA, (**C**) total lipid, and (**D**) ergosterol content of *P. italicum* mycelia at concentrations of 0, 1/2 MIC, MIC, and 2 MIC. Each value represents the mean ± SD. Means with different letters were significantly different at *p* < 0.05.

The extracellular conductivity of *P. italicum* was evaluated at 4, 8, 12, 24, 36, and 48 h after treatment with PCT nanoemulsions of different concentrations. As shown in Figure 4A, with an increasing exposure time, REC gradually increased for *P. italicum*. Within the first 12 h following treatment with PCT nanoemulsions, the value quickly increased, and the increase was noticeably greater than that of the control. From 12 to 36 h, although increasing trends occurred in all four groups, the REC values of the treatment group remained higher than that of the control group. After 36 h, the REC of the treatment group continued to increase at a slower rate, while that of the control group noticeably increased at the late stages, which may have been associated with the programmed cell death of *P. italicum*.

### 3.9. Malondialdehyde (MDA)

As shown in Figure 4B, MDA content was evaluated in *P. italicum* mycelia exposed to PCT at 0, MIC, 1/2 MIC, and 2 MIC for 5 days. MDA content was increased significantly by increasing PCT concentration, especially in the 2 MIC treatment group. In comparison with the control group (8.60 ± 0.77 nmol/g), the MDA contents of the 1/2 MIC, MIC, and 2 MIC PCT treatment groups were 15.31 ± 0.93, 18.15 ± 0.80, and 21.03 ± 0.94 nmol/g, respectively. The results showed that PCT treatment caused lipid peroxidation of *P. italicum*.

### 3.10. Total Lipid Content

Lipids are crucial structural substances of fungal membranes and play an important role in the bioactivity of cells [38]. As shown in Figure 4C, the total lipid content of *P. italicum* was different among the four groups, and it significantly decreased with increased PCT concentrations. The total lipid contents of *P. italicum* cells treated with 1/2 MIC, MIC, and 2 MIC of PCT were 279.7 ± 6.5, 178.67 ± 5.4, and 131.39 ± 8.6 g kg^−1^, respectively, and were considerably lower than that of the control (371.3 ± 7.6 g kg^−1^). The results indicated that *P. italicum* was strongly inhibited by PCT in the synthesis of cellular lipids.

### 3.11. Ergosterol Content

The fungus ergosterol regulates the enzymes involved in intracellular transport and maintains the integrity and permeability of the plasma membrane [39]. As shown in Figure 4D, the ergosterol content was dramatically reduced by adding PCT in a dose-dependent manner. The mycelial ergosterol content in the control was 0.36 ± 0.02 g kg^−1^, which was higher than that of the 1/2 MIC (0.27 ± 0.01 g kg^−1^), MIC (0.23 ± 0.02 g kg^−1^), and 2 MIC (0.14 ± 0.01 g kg^−1^) treatments, showing the PCT nanoemulsions highly inhibited the biosynthesis of fungal ergosterol.

## 4. Discussion

In previous studies, a large number of antimicrobial substances have been reported in citrus, such as flavonoids, essential oils, phenolic acids, and limonoids [25,40,41]. Among them, PMFs, a kind of flavonoid, widely exist in the citrus waste peel and have been regarded as a promising natural antimicrobial active substance in the food industry [42,43]. However, PMFs have the ability to inhibit *penicillium*, and their application is limited due to strong water insolubility and being less polar. In this study, PMFs and citral, two antimicrobial agents that are both derived from citrus, were encapsulated in a PCT nanoemulsion using nanotechnology. The nanoemulsion exhibited noteworthy antifungal activity against *P. italicum* by inhibiting mycelial growth and spore germination and destroying cell membrane structure. This research serves as a reference for exploring the use of citrus waste peel, understanding the potential antifungal mechanisms of fungicides, and developing novel antifungal compounds through the utilization of natural materials.

As reported previously, various essential oils such as eugenol, cinnamaldehyde, and thymol have the ability to inhibit fungal spore germination and mycelium growth [5,44], which is in agreement with our findings. Our results are encouraging when compared with those previously found by Tao et al. [45], who found the MIC value of free citral against *P. italicum* was 0.5 mL L^−1^, which is considerably higher than the value in this study. It is likely that PCT nanoemulsions have more effective antibacterial effects than lone citral due to nanoemulsions having better solubility and lower volatility. Additionally, combinations of volatile compounds and nonvolatile antimicrobial components may improve the effectiveness of antimicrobial agents and maintain their effectiveness for prolonged periods. In this study, adding PMFs increased the antibacterial activity of a citral emulsion; the MIC of the PCT nanoemulsion was 62.5 mg L^−1^, which was lower than that of the CT nanoemulsion. Our results are consistent with those reported by Li et al. [25], who found that limonin, a nonvolatile antimicrobial component, enhanced eugenol emulsion antifungal activity against *P. italicum*. In addition, a study on Salvia species also found that when the volatile and nonvolatile constituents were combined, the efficacy was enhanced [46].

The antifungal effect of essential oils has been shown to be exerted by influencing the integrity and permeability of the fungal cell membrane [47,48]. Therefore, we studied *P. italicum* treated with PCT under an electron microscope. SEM images (Figure 2) clearly showed that PCT resulted in abnormal appearances and a reduced number of spores and the irregular shrinkage and collapse of mycelia. These effects are similar to those of rosemary EO on *Aspergillus flavus* and tea tree EO on *Monilinia fructicola* [39,44]. The morphological changes in mycelia may have been due to increased cell permeability, which leads to the comprehensive leakage of intracellular substances, such as nucleic acids, proteins, and ions, or intercellular contents. These consequences are usually used to indicate severe and irreversible damage to the cell membrane [6,35]. As indicated by our results, the extracellular conductivity of the *P. italicum* supernatant rapidly increased after PCT treatment (Figure 4A). PI staining assays also demonstrated that the integrity and permeability of the cell membrane were damaged by PCT, which led to dye molecules entering the nucleus and emitting red fluorescence (Figure 3). These results are similar to those of [14], where citral and eugenol synergistically inhibited *Aspergillus Niger*, suggesting that the integrity and permeability of cell membranes had been impaired.

In addition, the increased permeability of the *P. italicum* cell membrane may have been due to lipid peroxidation, which accelerates cell apoptosis [49]. In this study, with nanoemulsions acting on *P. italicum* biofilms, the degree of cell membrane lipid peroxidation increased, resulting in an increase in the amount of MDA. This result is consistent with those of recent studies that investigated the effectiveness of paeonol [36], antofine [50], and zedoary turmeric oil [49] as antifungal agents against various fungal strains. This is also in agreement with a previous study by Yan et al. [51], who demonstrated that the action of antimicrobial components causes lipid peroxidation and structural damage within the cell membranes of microorganisms. Thus, the underlying antifungal action mechanism of PCT against *P. italicum* may have been a result of membrane lipid peroxidation, which changes the integrity and permeability of the cell membrane.

Lipids are essential constituents of the fungal cell membrane and perform various important biological functions [44,52]. In general, when lipid content decreases, membrane stability tends to be impaired and permeability to water-soluble materials becomes greater [53,54]. In this study, as a result of the addition of PCT, the lipid content of *P. italicum* was significantly decreased (Figure 4C). This result indicated that PCT destroyed the cell membrane structure and inhibited the growth of *P. italicum*. In addition, to ensure that PCT was targeting the plasma membrane, the amount of ergosterol was detected after PCT treatment. Ergosterol is an essential sterol for fungi and plays a crucial role in maintaining cell structure and function [44,55]. Commercial fungicides, such as imazalil, can greatly reduce the amount of ergosterol in fungal membranes by disrupting the normal sterol biosynthesis pathway [56]. In this study, PCT substantially impaired the biosynthesis of ergosterol in *P. italicum* cells (Figure 4D). These findings indicate that the plasma membrane may be a potential antifungal target of PCT. Although these results are encouraging, there are still many unknowns to be solved and explored, such as the low solubility of PMFs in citral and the in-depth antifungal mechanism of PCT against *P. italicum*. In particular, according to the results of this study, PCT exerts antifungal activity by disrupting the cell membrane of *P. italicum*; hence, a further study focusing on specific targets of PCT on fungal cell membranes may be carried out.

## 5. Conclusions

In this study, two antifungal ingredients, PMFs and citral, from citrus were prepared into a nanoemulsion with good stability and excellent antifungal activity against *P. italicum*. The potential antifungal mechanism was associated with the inhibition of fungal spore germination and mycelial growth and the destruction of cell membrane integrity and permeability. Therefore, this study lays a foundation for the development and utilization of antifungal ingredients from citrus and the control of *P. italicum* in citrus fruits.

## Figures and Tables

**Table 1:** Major chemical compounds of PMFs dissolved in citral.

	Compounds	Retention Time (min)	Concentration (g kg^−1^)
1	Isosinensetin	5.46	0.265
2	Sinensetin	5.60	0.571
3	3′,4′,5,7-Tetrathoxyflavone	5.78	0.067
4	Nobiletin	6.11	9.635
5	Tangeretin	6.45	8.488
	Total		19.026

**Table 2:** Characterization of nanoemulsions with different storage times at room temperature: evaluation of particle size, PDI, and zeta potential of nanoemulsions.

Storage Time (Days)	Particle Size (nm)	PDI	Zeta Potential (mV)
Fresh	17.08 ± 0.18 ^a^	0.215 ± 0.016 ^a^	−22.20 ± 0.80 ^a^
5	19.55 ± 0.25 ^b^	0.240 ± 0.021 ^a^	−15.15 ± 0.45 ^b^
10	20.71 ± 0.28 ^c^	0.219 ± 0.012 ^a^	−11.23 ± 1.27 ^c^
20	26.86 ± 0.96 ^d^	0.309 ± 0.003 ^b^	−11.05 ± 0.75 ^c^

Data are presented as means ± SDs. The room temperature was 25 °C. Different letters (a through d) indicate significant differences (*p* < 0.05) between values within the same column.

**Table 3:** MIC and MFC of CT and PCT nanoemulsions against *P. italicum*.

Group	Concentration ^a^	Mycelial Growth in PDB (3rd Day)	Mycelial Growth in PDB (6th Day)	MIC	MFC
CT	500	-	-	125	250
	250	-	-		
	125	-	+		
	62.5	+	+		
	31.25	+	+		
PCT	500	-	-	62.5	250
	250	-	-		
	125	-	+		
	62.5	-	+		
	31.25	+	+		
PDB + Stain	0	+	+		
PDB	0	-	-		

In the table, “+” represents visual mycelial growth and “-” represents no visual mycelial growth. ^a^ Concentration is expressed as mg L^−1^.

**Table 4:** Effect of different concentrations of a PCT nanoemulsion on spore germination of *P. italicum*.

Treatment	Spore Germination Rates		
	5 h	12 h	24 h
CK	4.91 ± 1.25 ^a^	53.45 ± 1.12 ^a^	91.69 ± 2.16 ^a^
1/2 MIC	0.93 ± 0.04 ^b^	25.56 ± 2.20 ^b^	48.09 ± 3.64 ^b^
MIC	0 ^c^	3.19 ± 0.16 ^c^	8.75 ± 0.40 ^c^
2 MIC	0 ^d^	0 ^d^	0 ^d^

Data are presented as means ± SDs. Different letters (a through d) indicate significant differences (*p* < 0.05) between values within the same column.

## Data Availability

The data presented in this study are available upon request from the corresponding author.
